# Data on fantasy vs contact driven internet-initiated sexual offences: Study selection, appraisal and characteristics

**DOI:** 10.1016/j.dib.2018.04.076

**Published:** 2018-04-25

**Authors:** L.J. Broome, C. Izura, N. Lorenzo-Dus

**Affiliations:** Swansea University, United Kingdom

## Abstract

Data presented within this article supports the findings of the manuscript “A systematic review of fantasy driven vs contact driven internet-initiated sexual offences: Discrete or overlapping typologies?” (Broome et al., in press) [1]. Inclusion and Exclusion criteria of study selection, PICO Formulation of Study Appraisal, as well as the Study Characteristics and Methodology of included studies are presented.

**Specifications Table**

**Table:** 

Subject area	*Psychology*
More specific subject area	*Forensic Behaviour*
Type of data	*Descriptive Tables*
How data was acquired	*Data extraction from studies included in Broome et al.**[1]*
Data format	*Descriptive*
Experimental factors	
Experimental features	*Data represents findings from a narrative review*
Data source location	*Reviewed studies collect data primarily from America, UK and Australia. It is not possible to determine specific states*
Data accessibility	*Within this article*

**Value of the data**

• The data allows for interpretation and assessment of studies examining the behavior of internet-initiated sexual crimes against minors, including study characteristics and methodology.
• The data enables comparison of two distinct classification of internet-initiated offences commonly referred to in the literature: fantasy vs contact driven crimes.
• Studies within this field primarily rely on the use of decoy victims, i.e. adults posing as children/young people who engage in proactive investigations. This data enables researchers to identify those studies that use decoy and real child victims.

## 1 Data

The data set contains information on the Inclusion and Exclusion Criteria for Study Selection (Table 1), and PICO Formulation of Study Appraisal (Table 2) for studies reviewed by Broome et al. [1]. Additionally, Study Characteristics (Table 3) and Methodology (Table 4) for included studies are described.

**Table 1:** Inclusion and Exclusion Criteria for Study Selection.

**Inclusion**	**Exclusion**
1. Studies that investigate the strategies of individuals who use the internet to sexually abuse minors.	Studies that investigate:
	a) offline sexual offending only b) child pornography use as a definition of ‘fantasy’ offending c) the sexual abuse of victims over 18 years of age d) offenders under 18 years of age
*and*	
2. Studies in which contact and fantasy driven behaviour were identifiable	
*and*	
3. Studies exploring crime characteristics or grooming strategies of individuals who use the internet to sexually abuse minors	
	Review articles and reports
*and*	
4. Primary studies to include cohort, case-control, cross-sectional or case series studies	Non-English articles

**Table 2:** PICO Formulation of Study Appraisal [2].

**PICO**	**Characteristics**
Population	Total number of participants (differentiating between fantasy and contact behaviour where appropriate), length of grooming/offending process, use of decoy victim.
Interest	Crime characteristics, behavioural tactics, communicative approaches and risk assessment strategies of adult online child sexual offenders. Consideration of typology (fantasy and contact groomers)
Comparisons	Evaluation of fantasy driven and contact driven individuals to assess whether an empirical distinction exists between the groups. Consideration of statistical analysis and study methodology.
Outcomes	Crime characteristics, analysis of tactics, communicative approaches and risk assessment strategies.

**Table 3:** Study Characteristics of Included Studies.

**Study**	**Typology**	**Offender Age**	**Offender Gender**	**Decoy Victim**	**Perceived Age of Victim**	**Victim Gender**	**Country of Data Source**	**N. Fantasy**	**N. Contact**	**Outcomes**
Barber [4]	Contact	Mean 35	#	Yes	Mean 13	#	America	–	90	Relationships, risk assessment, sexualisation, threatening behaviour, trade-off
Bergen [5]	Mixed	15^a^-60	*M*	Yes	10–18	#	Sweden/Finland	#	#	Trade-off
Bergen [6]	Mixed	Mean 25	*M* = 98	No	−13–17	*M* = 50	Sweden/Finland/Germany	66^a^	68^b^	Deception, trade-off
			*F* = 38			*F* = 80				
Black [7]	Contact	25–54	*M*	Yes	12–15	*M* = 6	America	–	44	Relationships, risk assessment, sexualisation, trade-off
						*F* = 38				
Briggs [8]	Mixed	19–54	M	No	12–16	*M* = 1	America	21	30	Deception, relationships, sexualisation, trade-off
						*F* = 50				
DeHart [9]	Mixed	18–74	M	Yes	9–14	*M* = 6	America	48 / 64^c^	44	Relationships, risk assessment, sexualisation, trade-off
						*F* = 194				
Grosskopf [10]	Mixed	#	*M*	Yes	13–14	*M*	Australia	5^d^	10^e^	Relationships, risk assessment, sexualisation, threatening behaviour, trade-off


**Study**	**Typology**	**Offender Age**	**Offender Gender**	**Decoy Used**	**Perceived Age of Victim**	**Victim Gender**	**Country of Data Source**	**N. Fantasy**	**N. Contact**	**Outcomes**
Gupta [11]	Contact	#	#	Yes	#	#	America	–	75	Relationships, risk assessment, sexualisation, trade-off
Kloess [12]	Mixed	27–52	*M*	No	11–15	*M* = 2	UK	3	2	Relationships, sexualisation, trade-off
						*F* = 3				
Krone [13]	Mixed	19–55	*M*	Yes	10–14	*F* = 23	Australia	8^f^	18^g^	Deception, relationships, risk assessment, sexualisation, trade-off
						*M* = 2				
Lorenzo-Dus [14]	Contact	22–63	*M*	Yes	–	–	America	–	24	Deception, relationships, risk assessment, sexualisation, threatening behaviour, trade off
Lorenzo-Dus [15]	Contact	22–63	*M*	Yes	–	–	America	–	68	Risk assessment, trade-off
Malesky [16]	Contact	23–52	*M*	No	#	#	America	–	31	Deception, trade-off
Marcum [17]	Contact	24–51	*M*	Yes	12–13	*F*	America	–	3	Deception, sexualisation, threatening behaviour, trade-off


**Study**	**Typology**	**Offender Age**	**Offender Gender**	**Decoy Use**	**Perceived Age of Victim**	**Victim Gender**	**Country of Data Source**	**N. Fantasy**	**N. Contact**	**Outcomes**
O’Connell [18]	Fantasy	#	#	Yes	8, 10 or 12	F	#	#	–	Deception, relationships, sexualisation, threatening behaviour, trade-off
Pranoto [19]	Contact	#	#	Yes	#	#	America	–	59	Relationships, sexualisation, threatening behaviour, trade-off
Quayle [20]	Contact	21–56	*M*	No	11–15	*M* = 1	Italy/UK	–	14	Deception, relationships, trade-off
						*F* = 13				
Shelton [21]	Contact	18–77	*M*	Yes	6–17	–	America	–	33^h^	Deception, trade-off
van Gijn-Grosvenor [22]	Contact	#	#	Yes	#	*M* = 49*F* = 52	America	–	101	Deception, relationships, sexualisation, trade-off


**Study**	**Typology**	**Offender Age**	**Offender Gender**	**Decoy Use**	**Perceived Age of Victim**	**Victim Gender**	**Country of Data Source**	**N Fantasy**	**N Contact**	**Outcomes**
Williams [23]	Contact	24–38	*M*	Yes	12–14	*F*	America	–	8	Deception, relationships, risk assessment, sexualisation, threatening behaviour, trade-off
Winters et al. [24]	Contact	19–64	*M*	Yes	12–15	*M* = 5	America	–	100	Deception, relationships, sexualisation, trade-off
						F = 95				
Wolak [25]	Mixed	18-40+	*M* = 2	No	12–17	*M* = 35	America	30	99	Deception, relationships, threatening behaviour, trade-off
			*F* = 127			*F* = 94				
Wolak [26]	Mixed	18-40+	*M* = 3	No	6–17	*M* = 15	America	68^i^	75^j^	Deception, threatening behaviour, trade-off
			*F* = 140			*F* = 128				

*Note.* Mixed typology refers to studies that report results for both contact and fantasy driven individuals. # Data not identifiable.

a Refers to those individuals who received a sexual picture (N=38) and engaged in cybersex (*N* = 28),

b Refers to those who met offline (*N* = 45) and engaged in offline sexual contact (*N* = 23).

c Includes cybersex and cybersex/schedulers.

d Represents individuals who engaged in cautious, more restrained exchanges (3) and educational exchanges (2).

e Refers to those who aim to reach short term sexual gratification (8) and long-term procurement (2).

f Signifies those individuals charged for exposing a child to indecent materials.

g Refers to individuals who procured a child online for sexual purposes.

h Represents traveling cases, including individuals who met victims online and travelled for intent of sexual contact.

i Includes individuals who committed a no contact offence.

j Includes contact offences (fondling, inappropriate touching (6), oral sex (10), intercourse (57) and sexual violence (2)).

**Table 4:** Methodology of included studies.

**Study**	**Methodology**	**Statistical Analysis**
**Barber et al.****[4]**	Content analysis from grounded theory and frequency word counts were carried out on online transcripts to assess for pervasiveness of communicative strategies.	–
**Bergen et al.****[5]**	Predictor variables of behaviour were coded based on expression of sexual interest from online transcripts	Contrast estimations and rank correlations were conducted to assess the level and direction of the effect of perceived age and behaviour. Inter-rater reliability was assessed with a median value of agreement being .69 (Cohen's K).
**Bergen et al.****[6]**	The prevalence and related outcomes of identity deception and keeping the online interaction a secret was assessed via online self-report surveys	Logistic regression analysis to examine the affect each item of deception and secrecy may have on outcomes. One-sample and independent t-tests were carried out on significant results. OR was reported for differences between the groups for each outcome. Bonferroni adjusted and non-adjusted p-values are reported.
**Black et al.****[7]**	Content analysis of grooming strategies, manually coded against the stages of O’Connell's (2013) proposed online grooming theory, was carried out on chat room transcripts. The Linquistic Inquiry Word Count (LIWC) was used to analyse several language categories representing different stages of grooming.	Mixed model analysis was carried out with language terms (friendship, relationship, risk assessment, exclusivity and sexual contact related terms) as the dependent variables and the grooming process stage as the independent variable. Chi-square analysis assessed specific manipulation techniques. Inter-rater reliability correlation of coding ranged from .34–.96 for frequency of use in strategies. Kappa values ranged from .72–.95 for presence of strategy.
**Briggs et al.****[8]**	Chat log transcripts were reviewed to identify communicative and behavioural patterns. Behavioural, social and clinical information was collected from archival data of individuals referred to a forensic mental health centre.	Cross-tabulation statistics were calculated to compare and contrast findings between contact and fantasy drive individuals.
**DeHart et al.****[9]**	Mixed method analysis of chat log transcripts were carried out to identify key elements of internet crimes against minors, proposing a typology of offenders. Qualitative coded was carried out using MaxQDA to sort commentaries into hierarchical categories.	Classification of offender type was based upon exploratory quantitative cluster analyses. Groups were compared using ANOVA for continuous variables and chi-square analysis for categorical variables.
**Grosskopf****[10]**	Semi-structures interviews were carried out with police officers involved in online sting operations to qualitatively compare findings to Krone [13]. For those unable to be interviewed, self-report questionnaires were distributed mirroring the questions asked in the interview	–
**Gupta et al.****[11]**	Word frequencies for each stage of O’Connell's [18] online grooming theory were calculated using LIWC and recorded in a 6 × 6 conditional probability matrix to calculate the probability of moving through each stage.	*Z*-scores for LIWC categories were calculated to normalise the data. Logistic regression analysis was carried out to calculate the linguistic predictor of each grooming stage. The variance inflation factor was calculated to assess multi-collinearity in the data and removing LIWC categories with an overlap of more than 80%.
**Kloess et al.****[12]**	Thematic analysis, employing a discursive content-driven approach was carried out on chat log transcripts to identify key information, trends and themes. A hierarchical grouping approach enabled assessment of similarity and differences across the categories	–
**Krone****[13]**	Police officers were interviewed and prosecution files were made available with access to demographics, previous criminal history and details about the arresting crime. Data was coded into a database. Both real victim and decoy victim data were included.	–
**Lorenzo-Dus et al.****[14]**	Using a Computer-Mediated Discourse Analysis approach, language –focused content analysis was carried out on chat logs. Focusing on speech acts and relational work, a new online grooming communicative model is proposed.	Welch's *t*-test was conducted to explore differences in the frequency of identified grooming processes. Pearson correlations examined relationships between grooming processes.
**Lorenzo-Dus and Izura****[15]**	A Computer-Mediated Discourse Analysis approach was undertaken. Praise was examined using Speech Act Theory (SAT – complimenting behaviour). The relational and procedural goals of groomers’ use of compliments were explored by the Interactional Sociolinguistics notion of relational work.	–
**Malesky****[16]**	Qualitative analysis was carried out on a questionnaire response to the question: “what initially attracted you to a particular child/adolescent online that you wanted to establish a relationship with for sexual purposes?” Participant responses were categorised into themes and evaluated by 3 independent reviewers.	–
**Marcum****[17]**	Qualitative latent coding on chat logs were carried out to explore the underlying meaning of the communication.	
**O’Connell****[18]**	Sociolinguistic analytical techniques were undertaken on grooming chat logs to develop a typology of child cybersexploitation	–
**Prantono et al.****[19]**	The term frequency-inverse document frequency (tf-idf) matrix was established from chat logs to identify grooming characteristics.	Paired *t*-tests were carried out to examine the relationship between words used and grooming characteristics. A logistic model was then developed using step-wise regression.
**Quayle et al.****[20]**	Using a constructivist grounded theory approach interview transcripts of convicted groomers where analysed to explore ways in which online groomers identified victims. Active language was analysed to explore categories within the data, inter-relationships between categories was assessed and theoretical sampling and sensitivity was incorporated into the analysis.	–
**Shelton et al.****[21]**	Investigative reports, offender interviews, sentencing information and criminal record information were accessed from FBI Crimes Against Children case reviews. Data extraction, to include offender background, investigation details and legal outcomes were recorded into an FBI developed protocol. The protocol was reviewed by the FBI's Behavioural Research Working Group.	Chi-square and t-tests were conducted to explore differences between cases that occurred between 1996 and 2002 (*n* = 198) and from 2010 (*n* = 53). No significant differences existed between the cases, the sample was therefore combined. A descriptive analysis of data extraction is provided.
**van Gijn-Grosvenor****[22]**	Qualitative coding of chat log transcripts was carried out by one researcher, identifying 4 categories describing groomer behaviour; offence characteristics, rapport building, sexual matters and concealment. Inter-rater reliability was measured on a sample of cases (*n* = 13), completed by a second coded. Coders agreed 93% of the time.	Chi square analysis and t-tests were carried out to examine differences between groomers targeting male and female victims.
**Williams et al.****[23]**	Chat logs transcripts were analysed thematically, in an inductive way, with no existing framework to code the data to identify grooming themes. One researcher carried out the initial coding, with a 2nd reviewed coding a sample (10%) of chat logs to evaluate consistency	–
**Winters et al.****[24]**	Inductive and deductive coding was carried out on chat log transcripts to investigate offender, decoy victim and conversation characteristics.	–
**Wolak et al.****[25]**	2574 Law enforcement agencies were surveyed and telephone interviews carried out to collect information about the case to include the type of crime, levels of deception, dynamics of the crime and type of sexual behaviour carried out	–
**Wolak et al.****[26]**	Law enforcement officers were interviewed using a computer-assisted telephone system following completion of a mail survey. Officers also provided a crime narrative. The overall aim of the study was to examine whether online groomers are a distinct offender group.	Chi-square cross-tabulation analysis was carried out to compare online-meeting and known-in-person cases. STATA SE11 survey data analysis procedures were employed to consider selection probability variations.

Note. – denotes data not applicable.

## 2 Experimental design, materials and methods

The process of study selection is defined in Table 1. The criteria were used to assess articles captured by the systematic search strategy in Broome et al. [1].

Studies included in Broome et al. [1] were appraised in consideration of the Population, Interest, Comparisons and Outcomes (PICO) formulation [2], and against an order of hierarchy regarding study methodology (Table 2). Data extraction was founded upon PRISMA guidelines [3] and piloted on a small sample of studies (*n* = 5).

Table 3 presents the Study Characteristics of included studies, to include Typology (contact, fantasy or mixed (i.e. both fantasy and contact behaviour)), Offender Age, Offender Gender, Decoy Victim, Perceived age of Victim, Victim Gender, Country of Data Source, Number of Fantasy Individuals, Number of Contact Individuals and Outcomes. Quantitative and Qualitative Methodological approaches for reviewed studies are presented in Table 4. Study Quality and Methodological appraisal is considered in Broome et al. [1].
